# Personalised blood pressure management during major noncardiac surgery and postoperative neurocognitive disorders: a randomised trial

**DOI:** 10.1016/j.bjao.2024.100294

**Published:** 2024-07-01

**Authors:** Julia Y. Nicklas, Alina Bergholz, Francesco Däke, Hanh H.D. Pham, Marie-Christin Rabe, Hanna Schlichting, Sophia Skrovanek, Moritz Flick, Karim Kouz, Marlene Fischer, Cynthia Olotu, Jakob R. Izbicki, Oliver Mann, Margit Fisch, Barbara Schmalfeldt, Karl-Heinz Frosch, Thomas Renné, Linda Krause, Christian Zöllner, Bernd Saugel

**Affiliations:** 1 Department of Anesthesiology, Center of Anesthesiology and Intensive Care Medicine, University Medical Center Hamburg-Eppendorf, Hamburg, Germany; 2 Outcomes Research Consortium, Cleveland, OH, USA; 3 Department of Intensive Care Medicine, Center of Anesthesiology and Intensive Care Medicine, University Medical Center Hamburg-Eppendorf, Hamburg, Germany; 4 Department of General, Visceral and Thoracic Surgery, University Medical Center Hamburg-Eppendorf, Hamburg, Germany; 5 Department of Urology, University Medical Center Hamburg-Eppendorf, Hamburg, Germany; 6 Department of Gynecology, University Medical Center Hamburg-Eppendorf, Hamburg, Germany; 7 Department of Trauma and Orthopaedic Surgery, University Medical Center Hamburg-Eppendorf, Hamburg, Germany; 8 Institute of Clinical Chemistry and Laboratory Medicine, University Medical Center Hamburg-Eppendorf, Hamburg, Germany; 9 Irish Centre for Vascular Biology, School of Pharmacy and Biomolecular Sciences, Royal College of Surgeons in Ireland, Dublin, Ireland; 10 Center for Thrombosis and Hemostasis (CTH), Johannes Gutenberg University Medical Center, Mainz, Germany; 11 Institute of Medical Biometry and Epidemiology, University Medical Center Hamburg-Eppendorf, Hamburg, Germany

**Keywords:** anaesthesia, cardiovascular dynamics, haemodynamic monitoring, individualised, morbidity, mortality, randomised controlled trial

## Abstract

**Background:**

It remains unknown whether there is a causal relationship between intraoperative hypotension and postoperative neurocognitive disorders. We tested the hypothesis that personalised—compared to routine—intraoperative blood pressure management reduces the incidence of postoperative neurocognitive disorders in patients having major noncardiac surgery.

**Methods:**

In this single-centre trial, 328 elective major noncardiac surgery patients were randomly allocated to receive personalised blood pressure management (i.e. maintaining intraoperative mean arterial pressure [MAP] above preoperative baseline MAP from automated 24-h blood pressure monitoring) or routine blood pressure management (i.e. maintaining MAP above 65 mm Hg). The primary outcome was the incidence of neurocognitive disorders (composite of delayed neurocognitive recovery and delirium) between postoperative days 3 and 7.

**Results:**

The primary outcome, neurocognitive disorders, occurred in 18 of 147 patients (12%) assigned to personalised and 21 of 148 patients (14%) assigned to routine blood pressure management (odds ratio [OR]=0.84, 95% confidence interval [CI]: 0.40–1.75, *P*=0.622). Delayed neurocognitive recovery occurred in 17 of 146 patients (12%) assigned to personalised and 17 of 145 patients (12%) assigned to routine blood pressure management (OR=0.99, 95% CI: 0.45–2.17, *P*=0.983). Delirium occurred in 2 of 157 patients (1%) assigned to personalised and 4 of 158 patients (3%) assigned to routine blood pressure management (OR=0.50, 95% CI: 0.04–3.53, *P*=0.684).

**Conclusions:**

Personalised intraoperative blood pressure management maintaining preoperative baseline MAP neither reduced the incidence of the composite primary outcome neurocognitive disorders between postoperative days 3 and 7 nor the incidences of the components of the composite primary outcome—delayed neurocognitive recovery and delirium—compared to routine blood pressure management in patients having major noncardiac surgery.

**Clinical trial registration:**

ClinicalTrials.gov (NCT03442907).

Postoperative neurocognitive disorders—including delayed neurocognitive recovery and delirium—are common^1^^,^^2^ and associated with long-term cognitive decline and death.^3^, ^4^, ^5^ The pathophysiology of postoperative neurocognitive disorders is multifactorial but presumably includes inadequate brain perfusion.^1^^,^^6^ Consistent with this theory, some studies show associations between intraoperative hypotension and postoperative neurocognitive disorders,^7^, ^8^, ^9^, ^10^ although others do not.^11^^,^^12^ It remains unknown whether there is a causal relationship between intraoperative hypotension and postoperative neurocognitive disorders.^13^^,^^14^

We thus sought to investigate whether targeted intraoperative blood pressure management reduces the risk of postoperative neurocognitive disorders. Preoperative baseline blood pressure varies substantially among individuals.^15^ We, therefore, assumed that intraoperative blood pressure intervention thresholds may best be defined based on individual preoperative baseline blood pressure.^16^^,^^17^ The optimal method to assess preoperative baseline blood pressure is automated ambulatory blood pressure monitoring.^18^, ^19^, ^20^, ^21^

We tested the hypothesis that personalised intraoperative blood pressure management maintaining preoperative baseline mean arterial pressure (MAP) from automated 24-h blood pressure monitoring reduces the incidence of neurocognitive disorders between postoperative days 3 and 7 compared to routine blood pressure management in patients having elective major noncardiac surgery.

## Methods

### Trial design

The single-centre randomised controlled ‘Intraoperative blood pressure Management based on the individual blood PRessure profile: impact on postOperatiVE organ function’ (IMPROVE) trial was conducted between 5 March 2018 and 10 October 2019 at the University Medical Center Hamburg-Eppendorf, Hamburg, Germany. The trial was approved by the ethics committee (Ethikkommission der Ärztekammer Hamburg, Hamburg, Germany) on 20 December 2016 (registration number PV5413) and all patients provided written informed consent. The trial was registered at ClinicalTrials.gov (NCT03442907) on 22 February 2018. The statistical analysis plan was written and approved by the principal investigators and trial statistician before data analysis. This trial is reported according to the Consolidated Standards of Reporting Trials statement.^22^

### Patients

We enrolled patients scheduled for elective major noncardiac surgery with general anaesthesia expected to last ≥90 min who were ≥50 yr old and classified as American Society of Anesthesiologists physical status class 2–4. We excluded patients who had a history of cerebrovascular events; had dementia; had kidney transplants; required dialysis; or were scheduled for emergency, cardiac, vascular, transplant, or neurosurgery.

### Preoperative automated 24-h blood pressure monitoring

To assess preoperative baseline blood pressure, all patients had preoperative automated 24-h blood pressure monitoring (boso TM-2430; Bosch+Sohn, Jungingen, Germany) for one day and night either at home or in the hospital. Blood pressure was measured every 15 min during the day and every 30 min at night. We only considered daytime blood pressures measured between 9:00 am and 9:00 pm, and nighttime blood pressures measured between 12:00 am and 06:00 am to exclude retiring and rising periods.^23^^,^^24^ Blood pressure measurements with a pulse pressure <20 mm Hg were discarded as artifacts, as were measurements that were marked as artifacts by the software used to read out the boso TM-2430 device (boso profil-manager XD; Bosch+Sohn). Patients with fewer than 4 daytime or nighttime blood pressure measurements dropped from the trial before randomisation.

### Randomisation and protocol

Patients were randomly allocated to receive personalised blood pressure management or routine blood pressure management in a 1:1 ratio without stratification using computer-generated codes. Allocation was concealed in consecutively numbered opaque envelopes until shortly before induction of anaesthesia.

We defined the preoperative baseline MAP individually for each patient as the mean of the mean daytime MAP and mean nighttime MAP. In patients assigned to personalised blood pressure management, clinicians were asked to maintain intraoperative MAP above the preoperative baseline MAP. We specifically asked clinicians to maintain MAP within a range of the preoperative baseline MAP+10 mm Hg (with a minimum MAP target range of 65–75 mm Hg and a maximum MAP target range of 100–110 mm Hg). Personalised blood pressure management started with the induction of anaesthesia and lasted through surgery. There was no specific haemodynamic management protocol, and clinicians used their discretion to maintain target MAP.

In patients assigned to routine blood pressure management, clinicians were unaware of the results of preoperative automated 24-h blood pressure monitoring and thus managed blood pressure per institutional routine which generally is to maintain MAP above 65 mm Hg.^25^^,^^26^

In all patients, general anaesthesia was maintained with inhaled sevoflurane or a continuous propofol infusion, and sufentanil boluses or a continuous remifentanil infusion. Patients' lungs were mechanically ventilated either via a tracheal tube or a laryngeal mask. Blood pressure was monitored using arterial catheters or upper-arm cuff oscillometry. When clinically indicated, epidural catheters were inserted before induction of general anaesthesia.

### Outcomes

The primary outcome was the incidence of neurocognitive disorders (collapsed composite of delayed neurocognitive recovery and delirium)^2^ between postoperative days 3 and 7.

As secondary outcomes, we assessed the incidence of each of the two components of the composite primary outcome. Additional secondary outcomes were the incidences of mild and major delayed neurocognitive recovery between postoperative days 3 and 7, changes in estimated glomerular filtration rate within the first 3 postoperative days, the incidence of acute kidney injury within the first 3 postoperative days, the incidence of acute myocardial injury within the first 3 postoperative days, the incidence of postoperative complications according to the European Perioperative Clinical Outcome definitions^27^ within the first 30 postoperative days, the hospital length of stay, and the incidence of all-cause mortality at postoperative days 30 and 90.

#### Postoperative neurocognitive disorders

We report postoperative neurocognitive disorders as a composite of delayed neurocognitive recovery and delirium according to a recent recommendation for the nomenclature of cognitive change associated with anaesthesia and surgery.^2^ In the trial protocol and trial registration, we used the old term *postoperative cognitive dysfunction* for what is currently referred to as *delayed neurocognitive recovery*.

To assess delayed neurocognitive recovery, we performed neuropsychological testing at least 1 day before surgery and once between postoperative days 3 and 7. Testing was conducted while patients remained hospitalised. Before surgery, we screened patients for depression with the Patient Health Questionnaire-9^28^ because depression might confound delayed neurocognitive recovery. Neuropsychological testing was performed with the computerised Wiener Test System (Schuhfried, Mödling, Austria). All tests were performed in a standardised manner by trained trial personnel. The test battery consisted of the Trail Making Tests A and B and the Stroop Color Word Interference Test. With the Trail Making Tests A and B, we tested cognitive processing speed and flexibility.^29^ In the Trail Making Test A, we evaluated the time patients took to connect numbers in consecutive order. In the Trail Making Test B, we evaluated the time patients took to connect numbers and letters in alternating order. With the Stroop Color Word Interference Test, we tested colour word interference tendency (Supplementary Data 1).^30^

We thus considered five cognitive variables: time for the Trail Making Test A, time for the Trail Making Test B, reading interference, naming interference, and processing time for the Stroop Color Word Interference Test. We calculated the reliable change index to define delayed neurocognitive recovery (Supplementary Data 1).^31^, ^32^, ^33^

Delirium was assessed using the confusion assessment method for the intensive care unit (CAM-ICU)^34^ once between postoperative days 3 and 7. When patients were still treated in the intensive care unit, we instead used intensive care delirium screening checklist (ICDSC) scores^35^ obtained for clinical purposes on an as-available basis. Any CAM-ICU score or ICDSC score indicating delirium between postoperative days 3 and 7 was considered evidence of delirium. Specifically, delirium was considered present when (a) the CAM-ICU identified acute change or fluctuating course of mental status and inattention, along with either disorganised thinking or an altered level of consciousness or (b) the ICDSC score was ≥4.

#### Changes in estimated glomerular filtration rate and acute kidney injury

We considered the most recent serum creatinine value before surgery as the preoperative baseline and the highest value observed within the first 3 postoperative days. We used the Cockcroft–Gault equation to calculate estimated glomerular filtration rate^36^ and calculated preoperative-to-postoperative estimated glomerular filtration rate changes. Acute kidney injury within the first 3 postoperative days was defined as (a) an increase in serum creatinine of ≥50% from preoperative baseline or (b) need for renal replacement therapy.^37^ Postoperative creatinine assessments were obtained for clinical purposes and used on as-available basis.

#### Acute myocardial injury

Acute myocardial injury was defined as an increase in high-sensitivity troponin T concentration within the first 3 postoperative days per definition of ‘myocardial injury and infarction associated with noncardiac procedures’ described in the Fourth Universal Definition of Myocardial Infarction (Supplementary Data 1).^38^ High-sensitivity troponin T was measured before induction of general anaesthesia and once within the first 3 postoperative days.

#### Postoperative complications, hospital length of stay, and all-cause mortality

Postoperative complications within the first 30 postoperative days were defined as any moderate or severe complication according to the European Perioperative Clinical Outcome definitions.^27^ All-cause mortality was assessed on postoperative days 30 and 90. Data were obtained from electronic health records and by phone interviews with patients who were discharged before postoperative day 30.

### Statistical analysis

Baseline and clinical characteristics are reported separately for patients assigned to personalised and to routine blood pressure management. Categorical data are presented as absolute number (percentage), and continuous data as median (25th–75th percentile). To describe baseline imbalances between the two treatment groups, we calculated absolute standardised differences^39^ defined as the absolute value of the difference among means or proportions divided by the pooled standard deviation. We considered characteristics with absolute standardised differences exceeding 0.2 as imbalanced.

We recorded intraoperative blood pressure at 5-min intervals. To quantify intraoperative blood pressure, we calculated the area under a MAP of 65 mm Hg. To quantify how well clinicians maintained intraoperative MAP above the preoperative baseline MAP, we calculated the fraction of time that intraoperative MAP was at the preoperative baseline MAP or higher, and the area under the preoperative baseline MAP. Areas under MAP thresholds were calculated by subtracting MAP measurements from MAP thresholds, multiplying positive differences with 5 min and adding those up.

Statistical analyses were performed according to the intention-to-treat principle. The level of statistical significance was 5% two-sided for the primary outcome. The primary outcome was analysed using a χ^2^ test and reported as odds ratio (OR) with 95% confidence interval (95% CI) based on the differences in the ratio of proportions. Missing data for the primary outcome were not imputed. For the primary outcome analysis, we considered patients in whom both neuropsychological testing (to assess delayed neurocognitive recovery) and delirium testing were available and patients who had either delayed neurocognitive recovery (but missing delirium testing) or delirium (but missing neuropsychological testing). We performed a primary outcome subgroup analysis for patients who were hypertensive according to preoperative automated 24-h blood pressure monitoring using an appropriate interaction test in a logistic regression model, in which the OR and 95% CI were based on the estimated marginal means. Hypertension was defined according to the European Society of Cardiology and the European Society of Hypertension guidelines for the management of arterial hypertension.^40^ In addition, we calculated the incidences of the primary outcome in patients assigned to personalised blood pressure management in whom the fraction of time that intraoperative MAP was at the preoperative baseline MAP or higher was ≥20%, ≥40%, ≥60%, and ≥80%. We also performed a *post hoc* subgroup analysis of patients aged <65 yr *vs* ≥65 yr using an appropriate interaction test in a logistic regression model, in which the OR and 95% CI were based on the estimated marginal means.

Binary secondary outcomes were analysed analogously to the primary outcome, if expected entries in the contingency table were smaller than 5, Fisher's exact test was used instead of a χ^2^ test; continuous secondary outcomes were analysed using a Wilcoxon rank-sum test with estimated difference in location and non-parametric 95% CI. Of note, the estimated difference in location does not provide an estimate of the difference in medians, but rather the median of differences between a sample from one treatment group and a sample from the other treatment group. For the secondary outcome analyses, no data imputation was performed and we only considered patients in whom the respective outcome data were available. Missing creatinine and troponin values were assumed to be normal.

We used R version 3.5.3 (R Foundation for Statistical Computing, Vienna, Austria) for statistical analyses.

### Sample size calculation

We assumed an incidence of neurocognitive disorders (collapsed composite of delayed neurocognitive recovery and delirium) between postoperative days 3 and 7 of 35% (adapted from our own data and the literature^41^). 138 patients per group (*n*=276 patients in total) would provide 80% power for detecting an absolute reduction in the incidence of the composite primary outcome from 35% in patients assigned to routine blood pressure management to 20% in those assigned to personalised blood pressure management at a significance level of 5% using a two-sided Z test with pooled variance. Allowing for 25% of the patients dropping out, we planned to enrol a total of 368 patients—184 patients per group.

## Results

**Figure 1:**
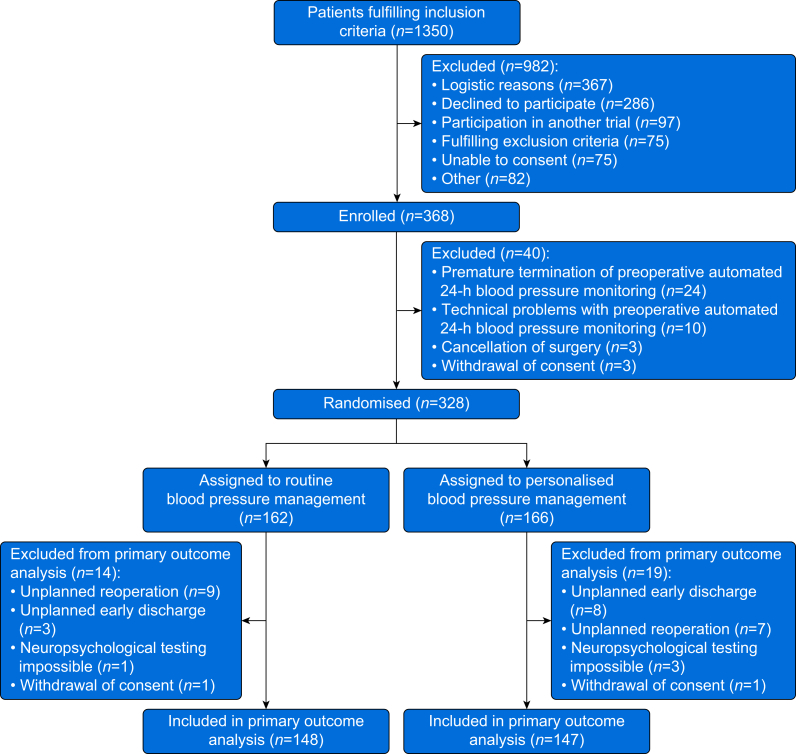
Patient flow chart. Flow chart illustrating patient screening, enrolment, randomisation, and reasons for exclusion.

We enrolled 368 patients but excluded 40 before randomisation (Fig 1). We thus randomly allocated 328 patients, 166 patients (51%) to personalised and 162 patients (49%) to routine blood pressure management (Table 1).

**Table 1:** Baseline and clinical characteristics. Categorical data are presented as number (percentage), continuous data as median (25th percentile–75th percentile). Baseline imbalances between the two treatment groups are presented using absolute standardised differences. Percentages may not sum up to 100% because of rounding. ∗Data were missing for 2 patients (1 patient assigned to routine and 1 patient assigned to personalised blood pressure management).

Characteristic	Routineblood pressuremanagement (*n*=162)	Personalisedblood pressuremanagement (*n*=166)	Absolutestandardiseddifference
**Age, yr**	66 (60–73)	66 (58–72)	0.040
**Height, cm**	172 (168–179)	174 (165–180)	0.011
**Weight, kg**	76 (69–90)	77 (65–90)	0.064
**Sex**			
Female, *n*	56 (35)	66 (40)	0.108
Male, *n*	106 (65)	100 (60)	
**American Society of Anesthesiologistsphysical status**			
2, *n*	83 (51)	99 (60)	0.180
3, *n*	78 (48)	65 (39)	
4, *n*	1 (1)	2 (1)	
**Baseline risk factors**			
Arterial hypertension, *n*	84 (52)	78 (47)	0.097
Antihypertensive medication, *n*	75 (46)	64 (39)	0.157
Chronic obstructive pulmonary disease, *n*	9 (6)	9 (5)	0.006
Coronary artery disease, *n*	21 (13)	11 (7)	0.214
Diabetes mellitus, *n*	33 (20)	19 (11)	0.246
Preoperative estimated glomerular filtration rate, ml min^−1^	85 (64–102)	83 (63–99)	0.043
Patients Health Questionnaire-9 score	5 (2–8)	4 (2–8)	0.067
**Automated 24-hblood pressure monitoring**			
At home, *n*	53 (33)	62 (37)	
In hospital, *n*	109 (67)	104 (63)	
Mean preoperative mean arterial pressure, mm Hg	94 (88–99)	95 (90–100)	
**Type of surgery**			
General surgery, *n*	64 (40)	70 (42)	
Urological surgery, *n*	50 (31)	54 (33)	
Gynaecological surgery, *n*	18 (11)	18 (11)	
Trauma surgery, *n*	14 (9)	13 (8)	
Otorhinolaryngology surgery, *n*	9 (6)	5 (3)	
Maxillofacial surgery, *n*	7 (4)	6 (4)	
Duration of surgery, min	168 (110–271)	180 (120–279)	
**Anaesthetic technique**			
Balanced anaesthesia, *n*	113 (70)	121 (73)	
Total intravenous anaesthesia, *n*	49 (30)	45 (27)	
Epidural block, *n*	86 (53)	83 (50)	
Intra-arterial blood pressure monitoring, *n*	113 (70)	126 (76)	
Cumulative volume of crystalloids, ml∗	2000 (1000–3000)	2500 (1500–3500)	
Cumulative volume of colloids, ml∗	0 (0–500)	0 (0–1000)	
Use of norepinephrine, *n*∗	156 (98)	165 (100)	
Norepinephrine dose, μg kg^−1^ min^−1^∗	0.07 (0.04–0.15)	0.15 (0.09–0.24)	
Use of inotropes, *n*∗	2 (1)	5 (3)	
Use of atropine, *n*∗	6 (4)	14 (8)	
Use of blood products, *n*	20 (12)	24 (14)	
Postoperative admission to intensive care unit, *n*	74 (46)	87 (52)	

**Figure 2:**
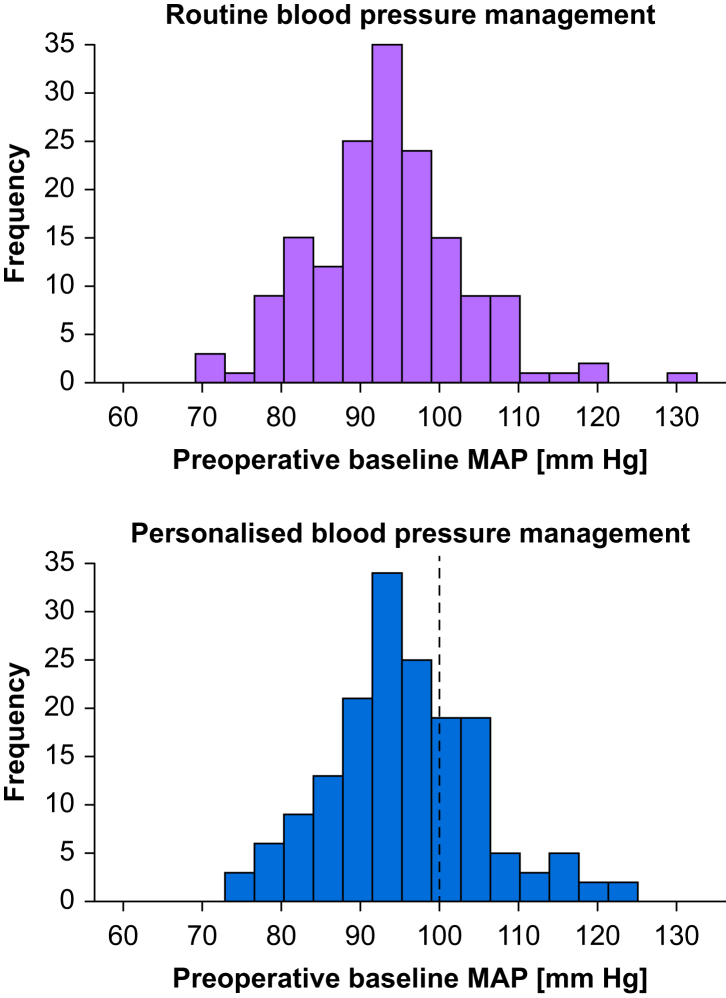
Preoperative baseline mean arterial pressure. Histograms showing the frequency of preoperative baseline mean arterial pressure (MAP) in patients assigned to personalised and routine blood pressure management. The dashed vertical line in the histogram showing patients assigned to personalised blood pressure management indicates the predefined maximum intraoperative MAP target of 100 mm Hg. MAP, mean arterial pressure.

A median of 16 (9–32) daytime and 12 (11–12) nighttime preoperative blood pressure measurements per patient were available. The median mean preoperative baseline MAP was 95 (90–100) mm Hg in patients assigned to personalised and 94 (88–99) mm Hg in patients assigned to routine blood pressure management (Fig 2). The mean preoperative baseline MAP varied substantially among individuals—and ranged from 71 to 131 mm Hg.

**Figure 3:**
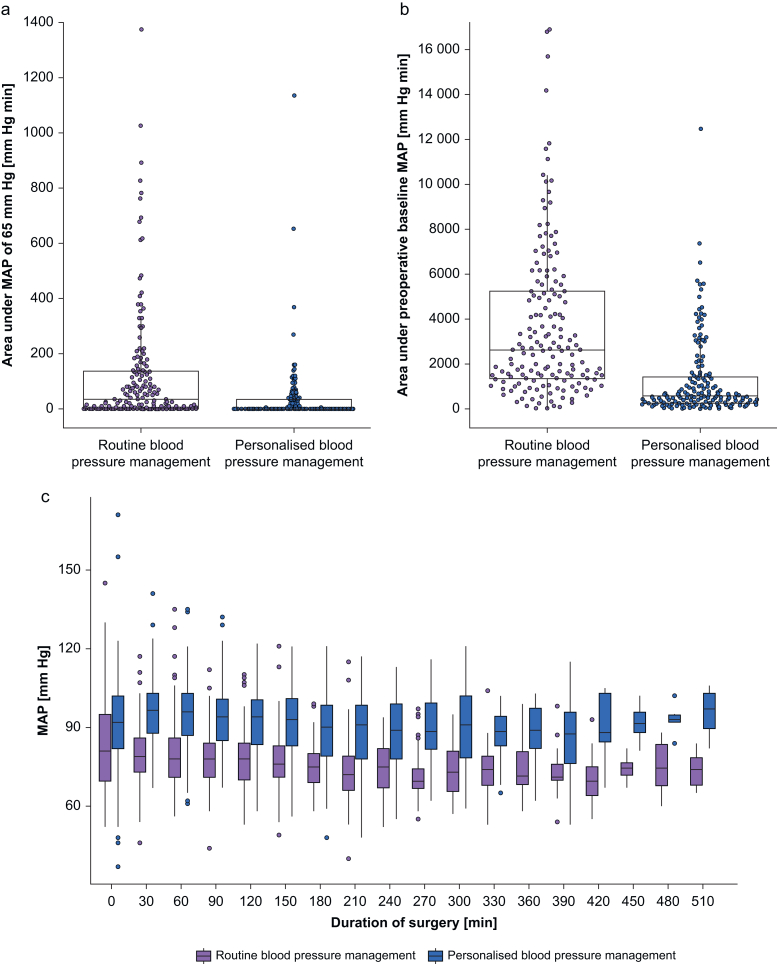
Intraoperative blood pressures. Boxplots with overlaying one dimensional scatter plots illustrating (a) areas under a mean arterial pressure (MAP) of 65 mm Hg, (b) areas under the preoperative baseline MAP, and (c) boxplots illustrating MAP every 30 min during surgery in patients assigned to personalised and routine blood pressure management. MAP, mean arterial pressure.

The median area under a MAP of 65 mm Hg was 0 (0–35) mm Hg min in patients assigned to personalised and 35 (0–138) mm Hg min in patients assigned to routine blood pressure management (Fig 3). The median fraction of time that intraoperative MAP was at the preoperative baseline MAP or higher was 57 (35–73)% in patients assigned to personalised blood pressure management, and 7 (2–24)% in patients assigned to routine blood pressure management. The median area under the preoperative baseline MAP was 563 (245–1408) mm Hg min in patients assigned to personalised blood pressure management and 2620 (1330–5204) mm Hg min in patients assigned to routine blood pressure management.

**Figure 4:**
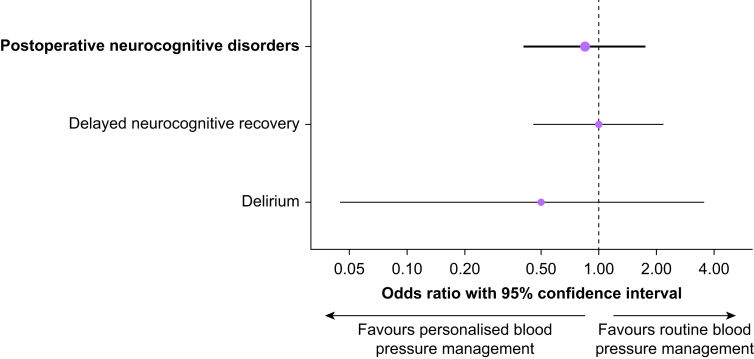
Primary outcome. Forest plots showing the effect of personalised compared to routine blood pressure management on the composite primary outcome and components.

The composite primary outcome neurocognitive disorders between postoperative days 3 and 7 occurred in 18 of 147 patients (12%) assigned to personalised and 21 of 148 patients (14%) assigned to routine blood pressure management (OR=0.84, 95% CI: 0.40–1.75, *P*=0.622; Fig 4; Table 2). Delayed neurocognitive recovery occurred in 17 of 146 patients (12%) assigned to personalised and 17 of 145 patients (12%) assigned to routine blood pressure management (OR=0.99, 95% CI: 0.45–2.17, *P*=0.983). Delirium occurred in 2 of 157 patients (1%) assigned to personalised and 4 of 158 patients (3%) assigned to routine blood pressure management (OR=0.50, 95% CI: 0.04–3.53, *P*=0.684).

**Table 2:** Primary and secondary outcomes. Categorical data are presented as number (percentage) with odds ratio (95% confidence interval), continuous data as median (25th percentile–75th percentile) with estimated difference in location (95% confidence interval). Percentages may not sum up to 100% because of rounding. EPCO, European Perioperative Clinical Outcome. ∗Data were missing for 33 patients (14 patients assigned to routine and 19 patients assigned to personalised blood pressure management). ^†^Data were missing for 37 patients (17 patients assigned to routine and 20 patients assigned to personalised blood pressure management). ^‡^Data were missing for 13 patients (4 patients assigned to routine and 9 patients assigned to personalised blood pressure management). ^¶^Data were missing for 24 patients (11 patients assigned to routine and 13 patients assigned to personalised blood pressure management). ^§^Data were missing for 5 patients (5 patients assigned to routine blood pressure management). ^||^Data were missing for 4 patients (4 patients assigned to routine blood pressure management).

Outcome	Routineblood pressuremanagement	Personalisedblood pressuremanagement	Odds ratio orestimateddifferencein location	*P*-value
Postoperative neurocognitive disorder, *n*∗	21/148 (14)	18/147 (12)	0.84 (0.40–1.75)	0.622
Delayed neurocognitive recovery, *n*^†^	17/145 (12)	17/146 (12)	0.99 (0.45–2.17)	0.983
Delirium, n^‡^	4/158 (3)	2/167 (1)	0.50 (0.04–3.53)	0.684
Mild delayed neurocognitive recovery, *n*^†^	9/145 (6)	12/146 (8)	1.35 (0.50–3.76)	0.507
Major delayed neurocognitive recovery, *n*^†^	8/145 (6)	5/146 (3)	0.61 (0.15–2.17)	0.412
Changes in estimated glomerular filtrationrate, ml min^-1¶^	10.2 (−2.8–21.6)	11.5 (−1.0–23.9)	−0.73 (−5.73–4.17)	0.741
Acute kidney injury, *n*	12/162 (7)	12/166 (7)	0.97 (0.39–2.45)	0.951
Acute myocardial injury, *n*	38/162 (24)	47/166 (28)	1.29 (0.76–2.19)	0.316
Postoperative complications accordingto EPCO definitions, *n*^§^	57/157 (36)	58/166 (35)	0.94 (0.58–1.52)	0.798
Hospital length of stay, days	10 (7–14)	11 (7–16)	−1.00 (−2.00–1.00)	0.261
Mortality at postoperative day 30, *n*^||^	3/158 (2)	0/166 (0)	0.00 (0.00–2.29)	0.115
Mortality at postoperative day 90, *n*^||^	5/158 (3)	6/166 (4)	1.15 (0.29–4.86)	1.00

In the subgroup of patients who were hypertensive according to preoperative automated 24-h blood pressure monitoring, postoperative neurocognitive disorders between postoperative days 3 and 7 occurred in 15 of 121 patients (12%) assigned to personalised and 16 of 106 patients (15%) assigned to routine blood pressure management (OR=0.80, 95% CI: 0.37–1.70, p_interaction_=0.835).

In patients assigned to personalised blood pressure management in whom the fraction of time that intraoperative MAP was at the preoperative baseline MAP or higher was ≥20%, ≥40%, ≥60%, and ≥80%, the incidences of postoperative neurocognitive disorders were 11% (14/126 patients), 10% (10/97 patients), 8% (5/67 patients), and 4% (1/22 patients), respectively.

We did not observe an effect of personalised—compared to routine—blood pressure management in the subgroup analysis of patients aged <65 yr *vs* ≥65 yr (p_interaction_=0.853). In patients aged <65 yr, the primary outcome (neurocognitive disorders) occurred in 3 of 67 patients (5%) assigned to personalised and 4 of 67 patients (6%) assigned to routine blood pressure management (OR=0.74, 95% CI: 0.26–3.43). In patients aged ≥65 yr, the primary outcome (neurocognitive disorders) occurred in 15 of 80 patients (19%) assigned to personalised and 17 of 81 patients (21%) assigned to routine blood pressure management (OR=0.87, 95% CI: 0.40–1.89).

There were no clinically important differences in any secondary outcome between patients assigned to personalised *vs* routine blood pressure management (Table 2).

## Discussion

In this trial, personalised intraoperative blood pressure management maintaining preoperative baseline MAP from automated 24-h blood pressure monitoring did not reduce the incidence of neurocognitive disorders in patients having elective major noncardiac surgery, although personalised blood pressure management resulted in substantially higher intraoperative blood pressures than routine blood pressure management.

There are only a few previous trials on targeted intraoperative blood pressure management^12^^,^^42^, ^43^, ^44^, ^45^ and only two specifically investigated postoperative neurocognitive disorders.^12^^,^^43^ The first was a pilot trial comparing the incidence of delirium within the first week after surgery or cognitive dysfunction at 3 months after surgery in 101 noncardiac surgery patients ≥75 yr old receiving personalised targeted or untargeted intraoperative blood pressure management.^12^ In patients assigned to personalised targeted intraoperative blood pressure management, clinicians aimed to keep intraoperative MAP at ≥90% of preoperative MAP (defined as the average of 3 MAP measurements performed at the pre-anaesthesia outpatient clinic).^12^ In patients assigned to personalised targeted—*vs* untargeted—intraoperative blood pressure management, the incidence of postoperative cognitive dysfunction was 11% *vs* 7% (relative risk: 1.52, 95% CI: 0.41–6.3; *P*=0.56) and the incidence of postoperative delirium was 6% *vs* 14% (relative risk: 0.44, 95% CI: 0.12–1.60; *P*=0.21).^12^ The results of the second previous trial—a multicentre trial in 322 noncardiac surgery patients aged ≥65 yr—suggested that targeted blood pressure management may reduce the incidence of postoperative delirium.^43^ Specifically, the incidence of postoperative delirium within the first week after surgery occurred in 25% of patients assigned to a MAP target of 60–70 mm Hg and in 12% of patients assigned to a MAP target of 95–100 mm Hg (relative risk: 0.48, 95% CI: 0.25–0.87; *P*=0.02).^43^

In our trial, we considered the theory that intraoperative blood pressure intervention thresholds may best be defined based on individual preoperative baseline MAP.^15^, ^16^, ^17^ Preoperative baseline MAP varied substantially across our patients—ranging from 71 to 131 mm Hg. Given this substantial interindividual variability in preoperative baseline MAP, our use of individualised instead of fixed intraoperative MAP targets seems reasonable.

To assess preoperative baseline MAP, we—for the first time in a perioperative trial—performed preoperative automated 24-h blood pressure monitoring. Automated ambulatory blood pressure monitoring presumably is the best method to assess preoperative baseline blood pressure^18^^,^^21^—because single blood pressure measurements before the induction of anaesthesia^15^ or in the clinic^46^ poorly reflect an individual's normal blood pressure. However, for organisational reasons, only a third of patients in our trial had preoperative automated 24-h blood pressure monitoring outside the hospital, whereas the others had it during hospitalisation. Additionally, not all patients were monitored for 24 h because we often started monitoring in the afternoon and continued until the next morning. We defined the preoperative baseline MAP as the mean of the mean daytime MAP and mean nighttime MAP. Nighttime blood pressure may better reflect the individual patients' blood pressure needed during surgery under general anaesthesia. In an ongoing follow-up multicentre trial,^47^ we therefore only consider nighttime MAP to define MAP intervention thresholds.

Protocol adherence is important in trials on targeted blood pressure management. In our trial, the median relative time the intraoperative MAP was at the preoperative baseline MAP or higher was 57% in patients assigned to personalised blood pressure management. However, the time above target only reflects for how long MAP was above or below the target MAP—but ignores to what extent the actual MAP was below the target MAP. We, therefore, also calculated the area under the preoperative baseline MAP. The median area under the preoperative baseline MAP was 563 mm Hg min in patients assigned to personalised blood pressure management—which, for example, would result when the actual intraoperative MAP was about 9 mm Hg below the target for 60 cumulative minutes, or about 3 mm Hg below the target for 180 cumulative minutes. The median target MAP was 95 mm Hg in patients assigned to personalised blood pressure management. Consequently, although clinicians were not able to always maintain MAP above the target MAP, there was a meaningful difference in intraoperative MAP between patients assigned to personalised *vs* routine blood pressure management. Additionally, profound hypotension was rare in patients assigned to personalised blood pressure management (the median area under a MAP of 65 mm Hg was 0 mm Hg min). In patients assigned to personalised blood pressure management, the incidence of postoperative neurocognitive disorders was lower when the fraction of time that intraoperative MAP was above the preoperative baseline MAP was higher. It remains speculative whether better protocol adherence would have changed our results.

A strength of our trial is that we performed complex neuropsychological testing for delayed neurocognitive recovery before and after surgery. The incidence of delayed neurocognitive recovery was 12%. However, the incidence of the primary composite outcome neurocognitive disorders (delayed neurocognitive recovery and delirium) was 13%—which was lower than we expected. In particular, the incidence of delirium was substantially lower than in previous studies where the reported incidence of delirium was between 5% and 52% depending on the type of surgery and patient baseline risk.^48^^,^^49^ The low incidence of delirium probably is a result of the pragmatic way we assessed delirium. We presumably often missed delirium because we screened for delirium only between postoperative days 3 and 7—and not on postoperative days 1 and 2. Additionally, we used the CAM-ICU test to screen for delirium in patients treated on normal wards. Optimally, we should have assessed delirium twice a day starting on the day of surgery using delirium screening tests specifically validated for patients being treated in intensive care units and on normal wards.^50^^,^^51^

The incidence of the primary outcome presumably would have been higher if we had restricted the trial to older patients who generally are at higher risk of developing delayed neurocognitive recovery and delirium.^52^^,^^53^ Additionally, it would have been interesting to assess delayed neurocognitive recovery later than a week after surgery—although this may have increased the risk of confounding by postoperative factors.

An additional limitation of our trial may be that blood pressure treatment was not managed according to a haemodynamic treatment protocol. Especially, there was no protocol for fluid therapy and clinicians mainly used norepinephrine to maintain MAP. Consequently, patients assigned to personalised blood pressure management were given twice as much norepinephrine as patients assigned to routine blood pressure management. We also did not measure the depth of anaesthesia in all patients. Further, randomisation was not stratified for type of surgery. Finally, this was a single-centre trial and presumably patient populations, haemodynamic management, and incidences of neurocognitive disorders will differ in other centres. Given that our trial has numerous limitations, robust large-scale trials are still needed to confirm that targeted blood pressure management does not reduce neurocognitive disorders.

In conclusion, in this trial, personalised intraoperative blood pressure management maintaining preoperative baseline MAP from automated 24-h blood pressure monitoring neither reduced the incidence of the composite primary outcome neurocognitive disorders between postoperative days 3 and 7 nor the incidences of the components of the composite primary outcome—delayed neurocognitive recovery and delirium—compared to routine blood pressure management in patients having elective major noncardiac surgery.

## Authors’ contributions

Trial conception and design: JYN, BeS.

Acquisition of data: AB, FD, HHDP, MCR, HS, SS.

Data analysis and interpretation: all authors.

Statistical analysis: JYN, AB, LK, BeS.

Drafting of manuscript: JYN, AB, LK, BeS.

Critical revision of article for important intellectual content: all authors.

Final approval of the version to be published: all authors.

Agreement to be accountable for all aspects of the work thereby ensuring that questions related to the accuracy or integrity of any part of the work are appropriately investigated and resolved: all authors.

## Funding

The trial was funded by the Else Kröner-Fresenius-Stiftung, Bad Homburg, Germany (2016_A200) and institutional or departmental sources.

## Declarations of interest

MoF is a consultant for Edwards Lifesciences (Irvine, CA, USA) and has received honoraria for consulting and giving lectures from CNSystems Medizintechnik (Graz, Austria). KK is a consultant for and has received honoraria for giving lectures from Edwards Lifesciences. KK is a consultant for Vygon (Aachen, Germany). MarlF receives research support from Medtronic (Minnesota, MN, USA) and from Pfizer Inc. (New York, NY, USA). BeS is a consultant for and has received institutional restricted research grants and honoraria for giving lectures from Edwards Lifesciences. BeS is a consultant for Philips North America (Cambridge, MA, USA) and has received honoraria for giving lectures from Philips Medizin Systeme Böblingen (Böblingen, Germany). BeS has received institutional restricted research grants and honoraria for giving lectures from Baxter (Deerfield, IL, USA). BeS is a consultant for and has received institutional restricted research grants and honoraria for giving lectures from GE Healthcare (Chicago, IL, USA). BeS has received institutional restricted research grants and honoraria for giving lectures from CNSystems Medizintechnik (Graz, Austria). BeS is a consultant for Maquet Critical Care (Solna, Sweden). BeS has received honoraria for giving lectures from Getinge (Gothenburg, Sweden). BeS is a consultant for and has received institutional restricted research grants and honoraria for giving lectures from Pulsion Medical Systems (Feldkirchen, Germany). BeS is a consultant for and has received institutional restricted research grants and honoraria for giving lectures from Vygon (Aachen, Germany). BeS is a consultant for and has received institutional restricted research grants from Retia Medical (Valhalla, NY, USA). BeS has received honoraria for giving lectures from Masimo (Neuchâtel, Switzerland). BeS is a consultant for Dynocardia (Cambridge, MA, USA). BeS has received institutional restricted research grants from Osypka Medical (Berlin, Germany). BeS was a consultant for and has received institutional restricted research grants from Tensys Medical (San Diego, CA, USA). BeS is an editor of the *British Journal of Anaesthesia*. JYN, AB, FD, HHDP, MCR, HS, SS, CO, JRI, OM, MargF, BaS, KHF, TR, LK, and CZ declare that they have no conflicts of interest.
