# Supplementary material for: Personalised blood pressure management during major noncardiac surgery and postoperative neurocognitive disorders: a randomised trial
Source: BJA Open. 2024 Jul 1;11:100294. doi: 10.1016/j.bjao.2024.100294 (PMC11267063; doi:10.1016/j.bjao.2024.100294)
Supplement: Multimedia component 1 [file mmc1.pdf]

Supplementary Data 1: Supplementary methods

Stroop Color Word Interference Test

Patients were asked to press coloured buttons (blue, green, red, or yellow) depending on colour words that were displayed in matching or non-matching colours. For example, when the word “red” was displayed in blue, patients were to press the red button. Patients were then asked to press the coloured buttons depending on the colour the colour words were displayed in. For example, when the word “green” was displayed in blue, patients were to press the blue button. Each of the two parts of the test consisted of 128 words. We considered the main test variables reading interference, naming interference, and processing time for the primary outcome analysis.

Calculation of the reliable change index

We calculated the reliable change index to define delayed neurocognitive recovery. The reliable change index (RCI) was only calculated when at least 2 of the 5 corresponding preoperative and postoperative cognitive variables were available (i.e., time for the Trail Making Test A, time for the Trail Making Test B, reading interference, naming interference, and processing time for the Stroop Color Word Interference Test). To calculate the RCI, we first subtracted the preoperative from the postoperative cognitive variable (for each of the 5 cognitive variables). We then calculated the means and standard deviations of these differences for patients assigned to routine blood pressure management for each variable. We subtracted this mean of the differences from each difference between preoperative and postoperative cognitive variables. We calculated Z-scores by dividing these differences by the standard deviation of the differences between preoperative and postoperative test variables for patients assigned to routine blood pressure management. Individual RCIs were calculated by

dividing the sum of the Z-scores of all 5 test variables for one patient by the standard deviation of the summed Z-scores of patients assigned to routine blood pressure management. Patients were considered to have delayed neurocognitive recovery when their RCI was ≥1. Mild delayed neurocognitive recovery was defined as an RCI ≥1 but <2, major delayed neurocognitive recovery was defined as an RCI ≥2.

Acute myocardial injury

Acute myocardial injury was defined as an increase in high-sensitivity troponin T concentration within the first 3 postoperative days per definition of “myocardial injury and infarction associated with non-cardiac procedures” described in the Fourth Universal Definition of Myocardial Infarction. Acute myocardial injury was thus defined as a postoperative troponin T concentration above the 99th percentile upper reference limit (14 ng/L) with a) a ≥60% increase from baseline when baseline troponin T concentration was ≤14 ng/L, or b) a ≥20% increase from baseline when baseline troponin T concentration was >14 ng/L.
